# Overnight exposure to pink noise could jeopardize sleep-dependent insight and pattern detection

**DOI:** 10.3389/fnhum.2023.1302836

**Published:** 2023-12-01

**Authors:** Beverly Vickrey, Itamar Lerner

**Affiliations:** Department of Psychology, The University of Texas at San Antonio, San Antonio, TX, United States

**Keywords:** sleep, memory consolidation, extraction of regularities, insight, pattern recognition, pink noise, 1/f noise, number reduction task

## Abstract

Accumulated evidence from the past decades suggests that sleep plays a crucial role in memory consolidation and the facilitation of higher-level cognitive processes such as abstraction and gist extraction. In addition, recent studies show that applying pink noise during sleep can further enhance sleep-dependent memory consolidation, potentially by modulating sleep physiology through stochastic resonance. However, whether this enhancement extends to higher cognitive processes remains untested. In this study, we investigated how the application of open-loop pink noise during sleep influences the gain of insight into hidden patterns. Seventy-two participants were assigned to three groups: daytime-wake, silent sleep, and sleep with pink noise. Each group completed the number reduction task, an established insight paradigm known to be influenced by sleep, over two sessions with a 12-h interval. Sleep groups were monitored by the DREEM 3 headband in home settings. Contrary to our prediction, pink noise did not induce an increase in insight compared to silent sleep and was statistically more similar to the wake condition despite evidence for its typical influence on sleep physiology. Particularly, we found that pink noise limited the time spent in the initial cycle of N1 just after sleep onset, while time spent in N1 positively predicted insight. These results echo recent suggestions that the time in the initial cycle of N1 plays a critical role in insight formation. Overall, our results suggest that open-loop pink noise during sleep may be detrimental to insight formation and creativity due to the alterations it causes to normal sleep architecture.

## Introduction

Substantial evidence suggests that sleep plays an active role in declarative memory consolidation and facilitates processes of abstraction, inference, and insight by supporting memory for “gist” during slow wave sleep (SWS; Squire, 1992; Plihal and Born, 1997; Wagner et al., 2004; Born et al., 2006; Fischer et al., 2006; Diekelmann et al., 2009; Rasch and Born, 2013; Lerner, 2017; Lerner and Gluck, 2018, 2019; Lerner et al., 2019). During SWS, hippocampal-dependent memories are integrated into the general knowledge structure in the neocortex, a process supported by temporal coupling between slow oscillations (SO; 0.5 – 1 Hz), hippocampal sharp wave ripples, and sleep spindles (Mölle et al., 2002; Mölle and Born, 2011; Abel et al., 2013; Chokroverty and Thomas, 2013; Walker and Robertson, 2016). Furthermore, during sharp wave ripples, recently stored experiences in the hippocampus are “played back” via sequences of neuronal firing in a compressed timescale, known as memory replay (Squire, 1992; Wilson and McNaughton, 1994; Buzsáki, 1996; McGaugh, 2000; Lee and Wilson, 2002; Squire et al., 2004; Diba and Buzsáki, 2007; Abel et al., 2013; Rasch and Born, 2013). Various models suggest replay is central to the transfer and integration of information from the hippocampus to the neocortex that yields memory consolidation (McClelland et al., 1995; Davidson et al., 2009; Gupta et al., 2010; Klinzing et al., 2019), with some recent models suggesting replay’s time-compressed nature plays a core role in the ability to formulate abstractions and gain insight into temporal regularities (Lerner, 2017; Lerner and Gluck, 2019, 2022; Lerner et al., 2019).

Studies have demonstrated that external factors can influence memory consolidation during sleep. Particularly, stimulation of slow oscillations during sleep enhances declarative memory retention and consolidation, increases slow wave activity (SWA), extends SWS, and boosts spindle activity (Marshall et al., 2006; Ngo et al., 2013; Papalambros et al., 2019). Various stimulation protocols have been employed, including Targeted Memory Reactivation (TMR), which attempts to intervene with particular memories by associating them with sensory stimuli during wake and then replaying those stimuli during sleep (Marshall et al., 2006; Rasch et al., 2007; Rudoy et al., 2009; Diekelmann et al., 2011); and less tailored stimulations, such as presentation of pink noise overnight, which does not require manipulations during wake (Marshall et al., 2006; Ngo et al., 2013; Ong et al., 2016; Leminen et al., 2017; Papalambros et al., 2017, 2019). Pink noise is comprised of all the frequencies within the range of human hearing (20Hz – 20kHz) with a power spectral density inversely proportional to the frequency, resulting in more intense lower frequencies, and is commonly applied in two ways: closed-loop, which involves online assessment of SOs and application of pink noise in conjunction with the oscillations, and open-loop, where pink noise is applied indiscriminately (Riedy et al., 2021). Pink noise applied during sleep is theorized to enhance and synchronize physiological processes through stochastic resonance, a phenomenon by which a signal is amplified by added noise, resulting in widespread changes that can affect memory consolidation (Gammaitoni et al., 1998; Zhou et al., 2012; Ngo et al., 2013; Ong et al., 2016; Kim and Whang, 2017; Leminen et al., 2017; Papalambros et al., 2019; Schade et al., 2020; Pinto, 2021). These changes involve alterations to sleep architecture, such as prolonged time spent in sleep stages N2 and SWS, decreased sleep stage latencies, and increases in SO synchronization, SO power, and SWA (Suzuki et al., 1991; Kawada and Suzuki, 1993; Zhou et al., 2012; Ngo et al., 2013; Ong et al., 2016; Kim and Whang, 2017; Leminen et al., 2017; Papalambros et al., 2017, 2019; Garcia-Molina et al., 2020; Schade et al., 2020).

While evidence suggests pink noise applied during sleep can affect the consolidation of simple memories like word-pair recall, its effect on more complex memory consolidation processes remains understudied (Ngo et al., 2013; Ong et al., 2016; Leminen et al., 2017; Papalambros et al., 2017, 2019). Sleep’s beneficial effect on cognitive functions such as abstraction, pattern detection, and gist extraction is associated with the same physiological components as those benefiting simple memorization, including SWS, SO, and spindles (Born et al., 2006; Rasch and Born, 2013). Since the beneficial effects of pink noise during sleep are hypothesized to rely on enhancements of these components, we can predict similar benefits for higher cognitive functions, although to our knowledge, this has not been previously tested.

The current study utilizes the number reduction task (NRT), a well-established paradigm for investigating insight and gist, to determine if continuous pink noise played during sleep aids in gaining insight into hidden regularities. The NRT contains an underlying temporal hidden rule that, if discovered, allows for a dramatic performance improvement. Importantly, reaction times (RTs) to task stimuli just before discovering the hidden rule slow down, potentially reflecting reprocessing of an early representation of the rule preceding insight (Wagner et al., 2004). Previous research shows that sleep significantly contributes to the likelihood of explicitly discovering the hidden rule, possibly by amplifying its early representation through compressed memory replay (Wagner et al., 2004; Lerner and Gluck, 2019). We hypothesize that applying open-loop pink noise during sleep would enhance its effect, leading to a higher probability of rule extraction and greater slowing of RTs just before insight occurs.

## Methods

### Participants and design

Eighty-seven undergraduate students from the University of Texas at San Antonio were recruited to participate for university course credit. Participants were randomly assigned to 3 experimental groups: wake, silent sleep (SS), and sleep with pink noise (PN). Fifteen participants were excluded due to failure to follow instructions during the first session (i.e., achieving less than 67% accuracy; *N* = 5), gaining insight during the exposure task (*N* = 1), or, in sleep groups, sleeping less than 4 h (*N* = 9). Data of the remaining 72 participants (Wake = 25; SS = 22; PN = 25) were analyzed (see Table 1).

**TABLE 1:** Demographic data, sleep and insight propensity of participants.

Variable	Wake	Silent sleep	Pink noise sleep
Sample size	*N* = 25	*N* = 22	*N* = 25
Gender (M / F)	11 / 14	8 / 14	9 / 16
Age	19.36 ± 1.96	19.68 ± 1.86	19.96 ± 2.67
Years of education	13.94 ± 1.79	13.84 ± 1.51	13.88 ± 1.92
**Ethnicity**			
W	5	5	3
H	10	11	13
B	2	2	3
A	3	1	3
I	0	0	0
Other	0	0	0
Biracial	5	3	3
Subjective sleep quality	–	3.03 ± 0.82	3.30 ± 0.61
TST	–	344.52 ± 72.07	378.56 ± 70.37
WASO	–	22.93 ± 18.87	17.76 ± 17.02
N1	–	20.60 ± 9.34	19.86 ± 7.73
%N1	–	5.75 ± 2.29	5.32 ± 1.93
N2	–	149.10 ± 30.28	181.78 ± 48.94
%N2	–	45.00 ± 6.75	47.60 ± 7.97
SWS	–	100.41 ± 33.70	102.29 ± 22.09
%SWS	–	29.14 ± 8.03	28.44 ± 7.05
REM	–	67.00 ± 25.57	80.34 ± 47.73
%REM	–	19.23 ± 5.37	18.80 ± 5.36
N1 Latency	–	30.36 ± 26.91	21.66 ± 22.41
N2 latency	–	36.33 ± 28.13	25.46 ± 22.12
SWS latency	–	47.74 ± 30.67	37.04 ± 24.80
**Insight – questionnaire**			
Gained (out of N)	0 / 25	4 / 22	1 /25
% Gained	0.00	18.18	4.00
**Insight – performance**			
Gained (out of N)	4 / 25	8 / 22	3/ 25
% Gained	16.00	36.36	12.00

Numbers above represent Mean ± Standard Deviation. M, males; F, females; W, White/Caucasian; B, African American/Black; A, Asian American; H, Hispanic/Latino American; I, Indian/Indigenous Alaskan; TST, total sleep time; WASO, wake after sleep onset; N1, minutes spent in stage 1 sleep; N2, minutes spent in stage 2 sleep; SWS, minutes spent in SWS; REM, minutes spent in REM sleep.

**FIGURE 1:**
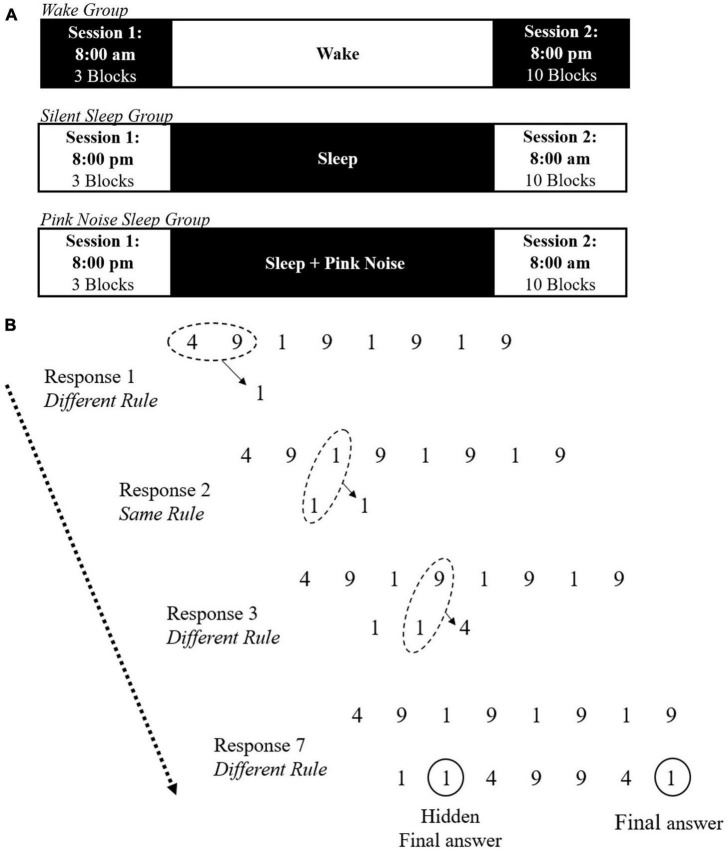
Illustration of the experimental task. **(A)** Timeline of the experimental procedure for the wake, silent sleep, and pink noise groups. **(B)** Example trial of the NRT including the same and different rules. On each trial, a string of eight digits is presented, composed of the digits 1, 4, and 9. For each input, participants must use the same and different rules to generate the next digit until reaching the final answer. The final response is always the same as the second one. Adapted from Wagner et al. (2004).

All participants were administered two sessions of the NRT task, “exposure” and “testing,” 12 h apart. The Wake group had their exposure session at 8:00 AM and testing session at 8:00 PM. SS and PN participants had their exposure session at 8:00 PM and testing session at 8:00 AM. All participants were required to avoid recreational drug or alcohol use during the study. The Wake group was instructed to continue their daily routine between sessions but refrain from sleeping. SS and PN participants were instructed to try sleeping for at least 8 h at home while monitoring their sleep with the DREEM 3 headband (Dreem, France), a mobile 4-channel (F7, F8, O1, O2) dry electrode EEG monitoring device fit for self-use (see Arnal et al., 2020 and Wood et al., 2023, for validation of the DREEM 3 automatic sleep staging algorithm accuracy compared to standard polysomnography as well as another mobile sleep monitoring device). Both sleep groups also completed a sleep log, reporting time in and out of bed, approximate sleep onset and wake time, and subjective sleep quality. Additionally, PN participants were provided with a pink noise machine (Adaptive Sound Technologies, Inc.) and instructed to place the machine approximately two feet from their bed and turn it on continuously at 55 decibels overnight at lights out, to avoid including activities before bed. The full experimental timeline is presented in Figure 1A.

### Experimental task

In the exposure session, participants completed three experimental blocks of the number reduction task (NRT) with 30 trials per block, modeled after Wagner et al. (2004; see Rose et al., 2002, for full details of this paradigm). In each trial, participants are presented with an eight-digit string made of 1, 4, and 9 (See Figure 1B). They are instructed to transform the given string into a new seven-digit string, one digit at a time, by following one of two rules: same rule and different rule. In each step, two digits are compared. If the digits are the same, they need to respond with the same digit (e.g., 4 and 4, yields 4). If the digits are different, they need to respond with the third digit (e.g., 4 and 9, yields 1). Participants compare the first two digits in the original string for the first response. From then on, the digits compared are the next digit in the original string and the previous response in the transformed string. The last (seventh) digit created is considered the answer for the trial, and participants indicate it by pressing a designated button. However, participants can indicate the final answer at any point during the trial by pressing this button after any digit in the sequence. Unknown to participants, the last three responses mirror the previous three in the formed string. Therefore, the second generated digit is always the same as the seventh generated digit. If participants become aware of this hidden rule, they can complete their second input and submit their final answer to end the trial and move on to the next one.

In the testing session, participants completed ten experimental blocks, each with 30 trials identical to the exposure session. After completing the trials, participants completed a questionnaire reporting and explaining whether they recognized any regularities in the task.

### Statistical analysis

Fisher’s exact test assessed insight gains among groups, with group as the independent variable and insight (Yes / No) as the dependent variable. Additionally, we compared RTs for those with and without insight. Following Wagner et al. (2004), participants’ responses were grouped into three categories: input 1, inputs 2–4, and inputs 5–7. We calculated RT differences between the last block of session one and first block of session two, reflecting any changes during the intermission period between sessions. A 2-way ANOVA was performed on RT differences for each group, with input (1, 2–4, 5–7) as a within-subject factor and insight (Yes/No) as a between-subject factor. Bonferroni-corrected pairwise comparisons followed significant effects and interactions.

For the two sleep groups, we first used *t*-tests to confirm that subjective sleep quality, total sleep time (TST), and wake after sleep onset (WASO) did not differ between groups. We then assessed sleep architecture variables that showed variations due to pink noise in previous studies, including time spent in N1, N2, and SWS, and N2 onset latency. Sleep staging was conducted automatically by the DREEM’s algorithms, and outliers (>2.5 standard deviations) were removed. One-tailed *t*-tests were conducted for each variable, reflecting the a-priori hypotheses based on previous studies that the value is higher (or lower, depending on the variable) in the PN group than in the SS group. We also examined potential relationships between each sleep variable and insight using stepwise binary logistic regression. The stepwise procedure began with an intercept-only model and used F-statistics *p*-values (entry: *p* < 0.09; remove term: *p* > 0.10) to select additional variables, allowing for statistical trends. A stepwise linear regression with a similar procedure examined relationships between each sleep variable and RT differences among input groupings.

## Results

Participants were categorized as having insight if they could describe the hidden rule in the post-experimental questionnaire and no insight if they could not. Four participants (18.18%) gained insight in the SS group, 0 in the wake group, and one (4%) in the PN group (Table 1). Using Fisher’s Exact Test, we first examined the a-priori hypothesis that sleep increases insight compared to wake, thus replicating the classic result with the NRT task. Results showed a trend-level effect (*p* = 0.09, one-tailed). However, when comparing all three groups (pink noise sleep, silent sleep, and wake), there were no significant differences in insight (*p* = 0.22). Conversely, the data showed a close numerical resemblance between the PN and wake groups, indicating a lack of typical insight-like improvements after sleeping in the PN group. Indeed, Fisher’s exact test failed to find any significant differences in insight between the PN and wake groups (*p* = 0.53). Merging the PN and wake groups and comparing it to the SS group revealed a marginal significance, indicating silent sleep resulted in more insight than the wake/PN groups combined (*p* = 0.07, one-tailed).

In previous studies (Wagner et al., 2004), participants who described the hidden rule consistently exhibited a shortening of response sequences during performance (i.e., supplying the answer to each trial within the first or second response after gaining insight). In our study, however, some participants exhibited shortening of sequences but could not verbally describe the hidden rule. When defining insight based on this sequence shortening performance rather than questionnaire responses, eight participants gained insight in the SS group (36.36%), four in the wake group (16.00%), and three in the PN group (12.00%). Thus, results showed a similar numerical pattern to questionnaire-based insight (Table 1). However, when re-running our analysis with this alternative measure of insight, Fisher’s exact tests showed no significance in any of the tests (all *p*’s > 0.19).

**FIGURE 2:**
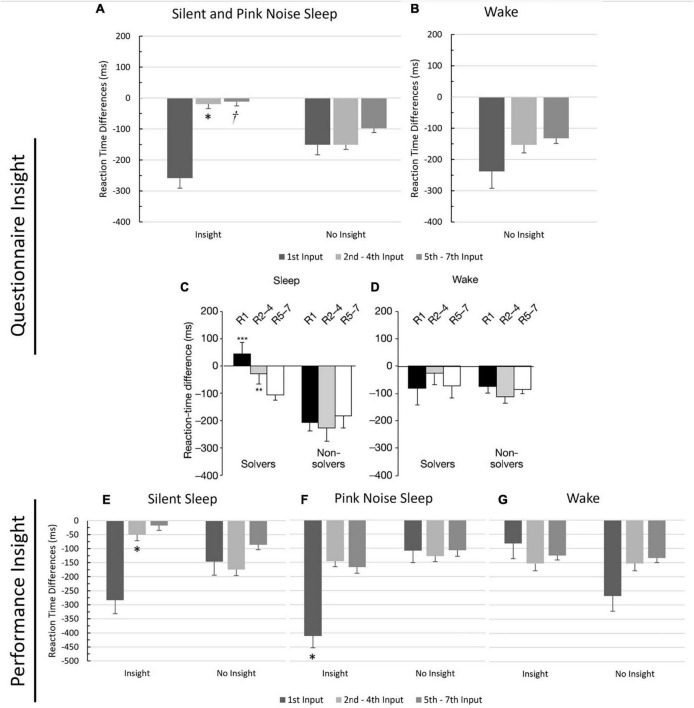
Comparison of reaction time differences between participants with insight and no insight. Values reflect the mean reaction time differences between the last block of Session 1 and the first block of Session 2 for each input type (1, 2–4, 5–7), grouped by insight. Means are reported on the *Y*-axis. Error bars reflect standard errors. *Significance at 0.05 level. †significance at the 0.09 level. **(A)** Silent and pink noise sleep groups using insight based on questionnaire responses. **(B)** Wake group using insight based on questionnaire responses (only non-insight included, no wake participants gained insight). **(C,D)** Reaction time differences per input for the sleep and wake groups from Wagner et al. (2004)’s NRT study, for comparison. “Solvers” indicates subjects with insight. Figures taken with permission from Wagner et al. (2004). **(E)** Silent sleep group using insight based on performance. **(F)** Pink noise sleep group using insight based on performance. **(G)** Wake group using insight based on performance.

Next, following Wagner et al. (2004), we ran a 2-way ANOVA to examine the effects of input order (1, 2–4, 5–7) and insight (Yes/No) on RT differences, using questionnaire-based insight (Figure 2). While Wagner et al. (2004) differentiated between wake and sleep groups in their analysis, we combined the two sleep groups and excluded the wake group since the PN group only had one participant who gained insight, and the wake group had none. Mauchly’s Test of Sphericity was violated, and the Greenhouse-Geisser correction was used. Overall, RTs were quicker in session two than in session one, demonstrated by a significant negative intercept (*p* < 0.001), indicating a general performance improvement. There was a significant effect of input order on the difference in RTs (*F*(1.29,77.45) = 4.92, *p* = 0.02, η^2^ = 0.08), with input 1’s difference being significantly larger than both input 2–4’s difference (*MD* = 119.00, *p* < 0.05) and input 5–7’s difference (*MD* = 150.00, *p* = 0.014). There was no significant effect of insight on RTs differences (*F*(1,60) = 0.44, *p* = 0.51, η^2^ = 0.01). Importantly, there was also a marginally significant interaction effect between input order and insight (*F*(1.29, 77.45) = 3.16, *p* = 0.07, η^2^ = 0.05), with participants exhibiting insight having significantly smaller RT differences for inputs 2–4 (*MD* = 131.59, *p* = 0.013), replicating previous results (compare Figures 2A, C), and marginally significant smaller RT differences for inputs 5–7 (*MD* = 85.91, *p* = 0.09) than those without insight.

The analysis was then repeated using performance-based insight. Each group had several participants who gained insight (Table 1), so we conducted separate analyses for each group to follow Wagner et al. (2004; Figure 2). Mauchly’s Test of Sphericity was violated in each group analysis, and the Greenhouse-Geisser correction was used. For the SS group, there was a significant main effect of input order on the difference in RTs (*F*(1.27, 40.49) = 5.82, *p* = 0.014, η^2^ = 0.15), which was qualified by a significant interaction between input order and insight (*F*(1.27, 40.49) = 4.08, *p* = 0.04, η^2^ = 0.11). Those with insight had a smaller RT difference on inputs 2–4 than those without insight (*MD* = −125.00, *p* = 0.02), once again replicating previous results. For the PN group, there was a marginally significant effect of input order on the difference in RTs (*F*(1.25, 32.48) = 3.02, *p* = 0.08, η^2^ = 0.10), a significant effect of insight on RT differences (*F*(1, 26) = 4.17, *p* < 0.05, η^2^ = 0.14), and a marginally significant interaction between input order and insight on the difference of RTs (*F*(1.25, 32.48) = 3.53, *p* = 0.06, η^2^ = 0.12). Participants with insight had larger RT differences on input 1 than those without insight (*MD* = 302.00, *p* = 0.02). For the wake group, there was no significant effect of input order on the RT differences (*F*(1.30, 29.87) = 0.23, *p* = 0.70, η^2^ = 0.01), no significant effect of insight on RT differences (*F*(1, 23) = 1.34, *p* = 0.26, η^2^ = 0.06), and no interaction effect between input order and insight on the RT differences (*F*(1.29, 29.87) = 1.17, *p* = 0.30, η^2^ = 0.05), again replicating previous results (Wagner et al., 2004).

Even in this last analysis using performance-based insight, the number of participants gaining insight in the PN and wake groups was still relatively low, adding uncertainty to the results. Since we previously showed that the two groups were similar in terms of insight behavior, we reran the ANOVA, merging the PN and wake groups. When analyzed together, there was no significant effect of input order on the RT differences (*F*(1.26, 64.271) = 1.35, *p* = 0.26, η^2^ = 0.03), no significant effect of insight RT differences (*F*(1, 51) = 0.34, *p* = 0.56, η^2^ = 0.01), and no interaction effect between input order and insight on RT differences (*F*(1.26, 64.271) = 0.06, *p* = 0.87, η^2^ = 0.001).

**FIGURE 3:**
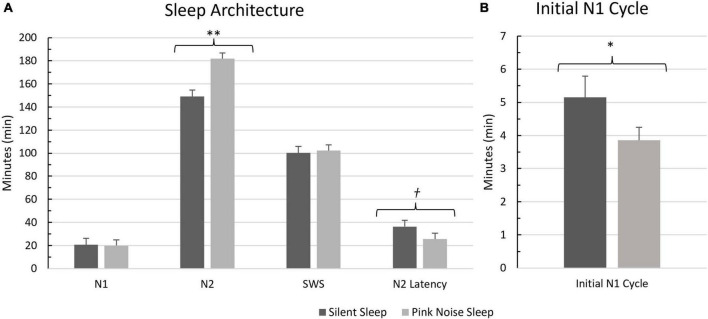
Comparison of sleep variables between the silent sleep and pink noise groups. Means and standard errors of each examined sleep variable are displayed. **(A)** Basic sleep architecture variables. **(B)** Time spent in the initial cycle of N1. ^**^Significance at 0.01 level, *significance at 0.05 level. †Significance at the 0.08 level.

Next, we tested a series of a-priori hypotheses examining how the application of open-loop pink noise during sleep modifies sleep physiology (Figure 3). Following previous literature, we hypothesized PN participants would spend less time in N1 (Kawada and Suzuki, 1993), more time in N2 (Suzuki et al., 1991) and SWS (Schade et al., 2020), and smaller latencies until N2 onset (Kawada and Suzuki, 1993) compared to SS participants. Replicating previous results, we found participants in the PN group spent significantly more minutes in N2 (*t*(44) = −2.66, *p* < 0.01, one-tailed) and had marginally significantly shorter latencies until N2 onset than the SS group (*t*(44) = 1.47, *p* = 0.08, one-tailed). There were no significant differences found between the groups in N1 percentage (*t*(43) = 0.68, *p* = 0.25) or minutes (*t*(44) = .29, *p* = 0.39), N2 percentage (*t*(45) = −1.20, *p* = 0.12), nor SWS percentage (*t*(45) = 0.32, *p* = 0.38) or minutes (*t*(44) = -.23, *p* = 0.41).

Although the difference between groups in time spent in N1 was not significant, the fact that N2 onset latency was marginally shorter in the PN group suggests pink noise might have reduced N1 early in the night since sleep typically begins with N1 and transitions to N2 as one falls into deeper sleep. Shortened N2 onset could thus imply a shortened initial cycle of N1. To confirm, we compared both sleep onset (which usually reflects N1 onset) and duration of the initial cycle of N1 between the groups. We found that sleep onset did not significantly differ between groups (*t*(44) = 1.20, *p* = 0.12); however, the duration of the initial cycle of N1 was significantly shorter for PN than for SS (*t*(43) = 1.80, *p* = 0.04, one-tailed).^1^ Therefore, PN did lead to reduced duration in N1, but only in the early cycle (Figure 3).

To follow up on this analysis, we used stepwise binary logistic regression to examine the potential relationship between the sleep variables of interest and questionnaire-based insight across all sleeping participants. Potential predictors included time spent in N1, N2, and SWS, time in the initial N1 cycle, and N2 onset latency. The stepwise procedure revealed that time in N1 was a significant positive predictor of insight (β = 0.188, *OR* = 1.21), indicating that the more minutes spent in N1, the greater the likelihood of insight. The model explained 36.5% of the variance in insight (χ^2^ = 6.60, *R^2^_*N*_* = 0.365, *p* = 0.01). We then reanalyzed the data using performance-based insight. N1 was again added to the final model with a positive coefficient (β = 0.137, *OR* = 1.15), indicating that the more minutes spent in N1, the greater the likelihood of insight. The final model explained 24.6% of the variance in insight (χ^2^ = 5.66, *R^2^_*N*_* = 0.246, *p* = 0.017). To follow up, we also conducted a point biserial correlation between the percent of N1 out of total sleep time and insight. There was a significant positive correlation between the proportion of time spent in N1 and insight with both measures of insight (questionnaire: *r*(45) = 0.377, *p* = 0.011; performance: *r*(45) = 0.310, *p* = 0.04). Participants with insight tended to have a greater proportion of N1.

Finally, we conducted a stepwise linear regression analysis to examine potential relationships between sleep variables and RT differences for each input grouping (1, 2–4, and 5–7). For input 1’s RT differences, the analysis resulted in the length of the initial N1 cycle being added to the final model with a negative coefficient (β = −33.95), indicating that the longer the initial N1 cycle, the larger the RT gap between sessions (see Figure 2). Additionally, time spent in N2 was included as a predictor, having a positive coefficient (β = 1.67), indicating that the more time spent in N2, the smaller the RT gap between sessions. The model explained 22.6% of the variance in RT differences (*F*(2, 38) = 5.55, *R*^2^ = 0.226, *p* < 0.01). For inputs 2–4, The final model was marginally significant, explaining 8.0% of the variance in RT differences (*F*(1,39) = 3.41, *R*^2^ = 0.080, *p* = 0.07) and included time spent in N1 as a predictor with a positive coefficient (β = 4.180). This result suggests that the more time spent in N1, the smaller the RT gap between sessions for inputs 2–4 (see Figure 2). In the final model using inputs 5–7, RT differences were not significant and no terms were added.

## Discussion

This study aimed to explore the effect of pink noise applied during sleep on the ability to gain insight into hidden regularities. Employing the NRT task, we showed that contrary to our predictions, pink noise did not lead to increased instances of insight. Instead, the pink noise sleep group was statistically more similar to the wake group, suggesting that the benefits for insight typically gained during a night of sleep were absent or counteracted by adding open-loop pink noise.

Time spent in N1 stood out as a potential cause of our unexpected results. More time and higher percentages of N1 predicted a higher likelihood of insight, and time spent in N1 was also associated with reduced RT differences for inputs 2–4, which are characteristic of participants gaining insight. Although overall differences in time spent in N1 between groups were not significant, the initial cycle of N1 was significantly shortened for the PN group. Recent findings support the significance of such shortened N1 during early sleep cycles by demonstrating that the initial N1 period after sleep onset is critical for solving the NRT as it represents a “creative sweet spot” (Lacaux et al., 2021). Spending as little as 1 min in N1 was associated in that study with a higher likelihood of hidden rule discovery. Considering our data, the PN group’s faster transition to N2 and reduced time in the initial N1 period likely diminished the potential of insight formation, resulting in fewer instances of insight than the SS group. The significance of the initial cycle of N1 could be due to the occurrence of hypnagogic content during this period (Ghibellini and Meier, 2023). However, hypnagogic content was not collected within this study, so support for this speculation is limited. On the other hand, SWS, which we initially hypothesized to contribute to elevated insight, was not affected by pink noise.

We replicated Wagner et al. (2004)’s findings showing a decrease in RT acceleration from the first to the second session for inputs 2–4, which was evident in sleeping participants who gained insight but not in those who did not gain insight or those who stayed awake. In our study, this exact result was observed for the SS and wake groups, with the PN group showing similar results to the wake group. Inputs 2–4 represent the first half of the underlying hidden pattern, which predicts upcoming responses. According to Wagner et al. (2004), slowed acceleration suggests the presence of an early representation interfering with implicit task performance; sleep amplifies the early representation, leading to greater interference, eventually overtaking the implicit memory representation and resulting in insight. The addition of open-loop pink noise may have eliminated the early representation’s amplification during sleep, potentially due to the decrease in early N1. Consistent with this interpretation, our stepwise linear regression confirmed that increases in N1 predict reduced acceleration in inputs 2–4, and, as noted above, instances of insight were correlated with higher levels of N1, regardless of how insight was defined.

A few of our findings are not readily explained. When measuring insight based on performance, there were indications that insightful participants exposed to pink noise showed a greater acceleration in RTs for input 1 than those who did not gain insight. Interestingly, this directly contrasted Wagner et al. (2004), where participants gaining insight after sleep had longer RTs for input 1 in their second session than the first. Our results also indicated that more time spent in N2 predicted a smaller acceleration in input 1’s RTs, and a longer initial N1 cycle predicted a larger acceleration in input 1’s RTs. While interpreting these findings is challenging, they seem unrelated to sleep-dependent insightful behavior. First, they relate to input 1 only, which is not part of the hidden structure. Second, the insight-based RT differences appear only when using performance-based rather than questionnaire-based insight. Third, N2, which predicted the RT differences, did not directly predict insight. Lastly, the effect vanished when the PN and wake groups were merged. Considering these factors and the inconsistency with the previous findings by Wagner et al. (2004), these results seem to be peripheral to our main aim and could stem from another, unspecified, cognitive process involved in how participants respond to input 1 in particular.

It is worth mentioning that only a few participants gained insight in our study, considerably fewer than previous NRT studies (e.g., Wagner et al., 2004; Yordanova et al., 2008; Lacaux et al., 2021). This may be due to our decision to emphasize ecological validity by having participants sleep in natural conditions at home and use noise machines in a straightforward, open-loop manner. However, this may have led to differences in individualized sleep schedules, a lack of a habituation night that may have led to first-night effects (e.g., Agnew et al., 1966), or a lack of control on activities, bedtimes, and waketimes between sessions. Future research on pink noise during sleep and its influence on high cognitive functions could focus on lab-based sleep monitoring, incorporating a habituation night to ensure better opportunities for insight gains, and closed-loop manipulations of pink noise. If home-based studies are pursued, they might incorporate rigid sleep and wake times with limitations on activities between sessions. Additionally, it should be noted that using a mobile sleep monitoring device for self-use, while allowing to maintain ecological validity, also has limitations. Specifically, the DREEM 3 employs a small number of dry channels rather than multiple wet channels like a standard polysomnography. As a result, reliably conducting in-depth analyses of the EEG signal, including spindle detection and power-band analysis, is difficult. Nevertheless, even when limiting the analysis to the validated sleep staging algorithm of the device, our results were largely consistent with previous literature on sleep-dependent insight and the physiological effects of pink noise.

Finally, it is important to consider the potential implications of our results for policies designed to protect sleep through the usage of pink noise or noise masking. Although pink noise aided in shortening the initial transitional period of sleep, which may help those who struggle attaining deeper sleep, it negatively affected higher-level cognitive functions. The cost-benefit balance of sleeping with pink noise should therefore always be taken in consideration. Overall, our results raise questions about the cognitive advantages attributed to exposure to open-loop pink noise during sleep, showing that it could lead to detrimental effects on insight and creativity.

## Data availability statement

The raw data supporting the conclusions of this article will be made available by the authors, without undue reservation.

## Ethics statement

The studies involving humans were approved by the UTSA Institutional Review Board. The studies were conducted in accordance with the local legislation and institutional requirements. The participants provided their written informed consent to participate in this study.

## Author contributions

BV: Conceptualization, Formal analysis, Writing – original draft. IL: Conceptualization, Methodology, Supervision, Writing – review and editing.

## Footnotes

1 One subject who transitioned from wake directly to N2 was removed from this analysis.
